# microProtein Prediction Program (miP3): A Software for Predicting microProteins and Their Target Transcription Factors

**DOI:** 10.1155/2015/734147

**Published:** 2015-04-28

**Authors:** Niek de Klein, Enrico Magnani, Michael Banf, Seung Yon Rhee

**Affiliations:** ^1^Carnegie Institution for Science, Department of Plant Biology, 260 Panama Street, Stanford, CA 94305, USA; ^2^Department of Genetics, University Medical Center Groningen, CB50, P.O. Box 30001, 9700 RB Groningen, Netherlands; ^3^Institut Jean-Pierre Bourgin, INRA Centre de Versailles-Grignon, route de St-Cyr (RD10), 78026 Versailles Cedex, France

## Abstract

An emerging concept in transcriptional regulation is that a class of truncated transcription factors (TFs), called microProteins (miPs), engages in protein-protein interactions with TF complexes and provides feedback controls. A handful of miP examples have been described in the literature but the extent of their prevalence is unclear. Here we present an algorithm that predicts miPs and their target TFs from a sequenced genome. The algorithm is called miP prediction program (miP3), which is implemented in Python. The software will help shed light on the prevalence, biological roles, and evolution of miPs. Moreover, miP3 can be used to predict other types of miP-like proteins that may have evolved from other functional classes such as kinases and receptors. The program is freely available and can be applied to any sequenced genome.

## 1. Introduction

Rearrangements in gene architecture are a driving force behind the evolution of novel functions in biology [1]. Genes can acquire novel genetic information by the reshuffling of existing genetic modules or the incorporation of novel ones [1]. Interestingly, the loss of coding sequence in a gene can also lead to important novel functions. A paradigm of truncated transcription factors (TFs), referred to as microProteins (miPs), is emerging in transcriptional regulation [2, 3]. miPs carry a protein-protein interaction domain that allows them to take part in TF complexes but lack the DNA binding domain (DBD). miPs might have evolved either through domain loss or by alternative transcription or splicing of TFs [2]. Alternatively, miPs might have arisen by convergent evolution, independent of TFs. All miPs described to date share sequence similarity with and are likely homologous to TFs. We refer to such TFs as miP target TFs. A miP can affect the function of its target TF by physically interacting either directly with its target TF (we classify these as direct target TFs) [4, 5] or with a partner of the target TF (indirect target TFs) [6, 7]. Several miPs have been shown to titrate their target TFs into an inactive form [4–7], while others work as cofactors in active protein complexes [8]. To date, miPs have been implicated to regulate developmental programs, hormone signaling, the circadian clock, and stress response pathways in metazoans and plants [2, 3]. Notwithstanding a few examples in the literature, the miP layer of transcriptional regulation is largely unknown.

Here we present the miP prediction program (miP3), a software that predicts miPs and their putative target TFs from a sequenced genome. The miP3 algorithm has been designed based on the properties of characterized miPs and exploits sequence similarity between miPs and target TFs for their detection.

## 2. Materials and Methods

### 2.1. miP3

miP3 is a command line program that predicts microProteins from a sequenced genome. It is implemented in Python. As input, it needs a FASTA file with all proteins in a given genome, a FASTA file with a class of proteins for which miPs are to be identified, for example, transcription factors, and a file with a list of unwanted domains, for example, DNA binding domains. To lower runtime, it makes use of the local BLAST+ tools [9]. As input, miP3 takes FASTA-formatted TF sequences to query against a database of proteins from a genome using BLASTP and a list of DNA binding domain IDs from Interpro database [10]. After the initial BLAST searches, a list of potential miPs is returned in a FASTA-formatted file. The putative miPs and their target TFs are subjected to InterproScan [11] to map protein domains. Putative miPs that are larger than 1.1 times the length of their target TFs are filtered out. Putative miPs that have DNA binding domains or domains that are not found in any of their target TFs are also filtered out. The putative miPs that have not been filtered out are written into a tab-delimited file containing the predicted miPs, their target TFs, domains they contain, and their protein lengths.

The version described here (version 2) has been improved from version 1 by removing the dependency of a locally installed InterproScan, using default parameters determined by a more thorough performance testing, and a number of other improvements detailed in the README (https://dpb.carnegiescience.edu/sites/dpb.carnegiescience.edu/files/readme_miP3V2.txt).

### 2.2. Availability of Supporting Data

The code is freely available at https://dpb.carnegiescience.edu/labs/rhee-lab/software. The software is distributed under the GNU General Public License (version 3 or later). Additional documentation is available from https://dpb.carnegiescience.edu/sites/dpb.carnegiescience.edu/files/readme_miP3V2.txt.

## 3. Results and Discussion

**Figure 1:**
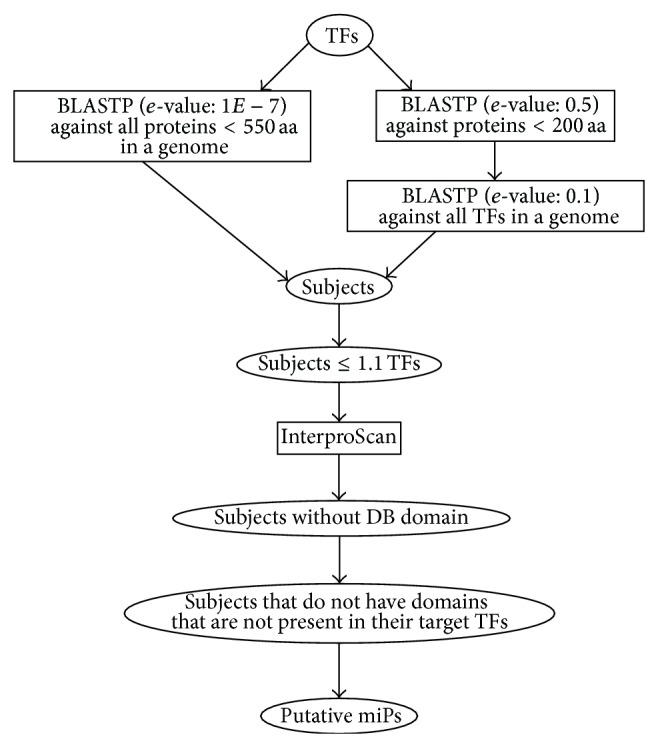
Diagram of miP3 showing all BLAST searches and filters used.

The miP3 algorithm detects putative miPs through sequence similarity with TFs and uses a number of filters to discard potential false positives. The algorithm is summarized in a diagram (Figure 1) and Pseudocode 1. Two types of BLAST searches are performed to identify putative miPs that share sequence similarity with TFs. First, a file containing TF sequences of an organism is used by miP3 as query in a BLASTP search against all proteins shorter than 550 amino acids in the genome with a default *e*-value cut-off of 1e-7. The protein length filter has been set at 550 amino acids because all miPs characterized to date and the average size of protein-protein interaction domains are smaller [12]. To our knowledge, the* Arabidopsis thaliana *LITTLE SIPPER is the largest miP (541 aa) characterized to date [2]. The *e*-value cut-off was determined empirically by testing different cut-off values against a set of known miPs in* A. thaliana* (see Supplemental Tables  1 and 2 in Supplementary Material available online at http://dx.doi.org/10.1155/2015/734147).

Second, because miPs are relatively short proteins (typically smaller than 200 amino acids), the TF sequences are also searched against proteins shorter than 200 amino acids at a lower stringency (default *e*-value cut-off of 0.5). In this second BLAST search, we opted for a higher *e*-value because the length of the protein found in a BLAST search is inversely proportional to the *e*-value. To reduce the number of false positives due to the less stringent *e*-value, the resulting hits are then filtered by a reverse BLASTP search against the TFs (default *e*-value cut-off of 0.1), and hits that do not match TFs are discarded. The efficacy of such a strategy has been already tested before [13] and proved successful in the miP3 algorithm. The *e*-values of these searches were empirically set to the most stringent values at the highest recall rate (Supplemental Tables  1 and 2).

The resulting set of putative miPs are subjected to a series of filters based on intrinsic and experimentally validated miP features. First, because miPs are TF-like proteins lacking a DNA-binding domain by definition, the algorithm discards putative miPs that contain DNA-binding domains through an InterproScan search [14]. Second, miPs are truncated TFs and hence they are smaller and bear fewer protein domains than their target TFs. Nevertheless, some miPs might have evolved from imprecise gene duplication and reshuffling events that incorporated novel unconserved DNA sequences causing miPs to be similar in size as their target TFs. To leverage this information, miP3 removes putative miPs that are longer than their target TFs with a 10% size tolerance. The 10% size tolerance was set to allow the inclusion of miPs that carry longer linker regions while excluding proteins that are much larger than the TFs and less likely to be miPs. miP3 also removes putative miPs that are predicted to carry domains different from their target TFs through the InterproScan software [11].

The performance of this version of the miP3 program was tested in* Arabidopsis thaliana*. To determine the best set of *e*-value thresholds, we compared a number of combinations of *e*-value thresholds for the three BLAST runs against a set of characterized miPs in* A. thaliana* (Supplemental Tables  1 and 2). We used the most stringent set of *e*-value thresholds at the highest recall rate (59% recall) as default parameter values in the software. The software set with the default parameters detected 10 of the 17 characterized miPs in* A. thaliana* (Supplemental Table  2). The false negatives fall into three categories (Supplemental Table  2): (1) one characterized miP does not have a protein sequence available in TAIR [15]; (2) one characterized miP was too divergent in sequence from the TFs; (3) 5 atypical bHLH miPs contain the HLH domain but are missing the basic residues, which is currently not detectable by domain mapping using InterproScan. It is difficult to assess precision without extensive experimental validation, which is beyond the scope of this paper. However, if we use physical interaction between a miP and its target TF as a criterion for being a true positive, we can assess precision based on the status of physical interactions of predicted miPs that have not yet been experimentally characterized. Five putative miPs that were predicted by miP3 to target* A. thaliana* homeodomain transcription factors were previously tested for physical interaction with their target TFs in yeast two-hybrid assays (Supplemental Table  2 and [2]). If we consider the three predicted miPs that failed to interact physically with the target TFs as false positives, the precision would be 40%. However, this is likely to be an underestimation because a number of characterized miPs have been shown to interact only with a partner of their target TFs and not with the target TFs themselves [2]. Currently the miP3 program relies on protein domain mapping based on the domain profiles available in the Interpro database, which can miss some miPs. For example, if a protein has sufficient sequence similarity to resemble a DNA binding domain but has other characteristics that prevent it from binding to DNA (e.g., missing basic residues in the bHLH domain), the protein will not be detected as a miP. Additional constraints that can distinguish functional DNA binding domains from nonfunctional DNA binding domains could help identify more miPs in the future.

## 4. Conclusions

miP3 has been designed to detect miP/TF couples that share sequence similarity. All miPs characterized to date are homologous to their target TFs. Nevertheless, we cannot exclude the existence of miP/TF couples that might have diverged considerably in sequence or evolved through convergent evolution. The software will help shed light on the prevalence and evolution of a potentially universal miP function. Moreover, the design of the software allows the prediction of any group of proteins that have evolved from different types of proteins by domain loss. For example, to search for proteins that are similar to kinases but have lost the kinase domain in a genome, one simply needs to replace the TF sequences with kinase sequences and the DNA binding domain list with a kinase domain list. The software is freely available and can be applied to any sequenced genome.

## Supplementary Material

The Supplementary Material contains two tables. Supplemental Table 1 compares the performance of miP3 with different parameter values. Supplemental Table 2 lists known or putative miPs that were used for performance assessment

## Figures and Tables

**Pseudocode 1:**
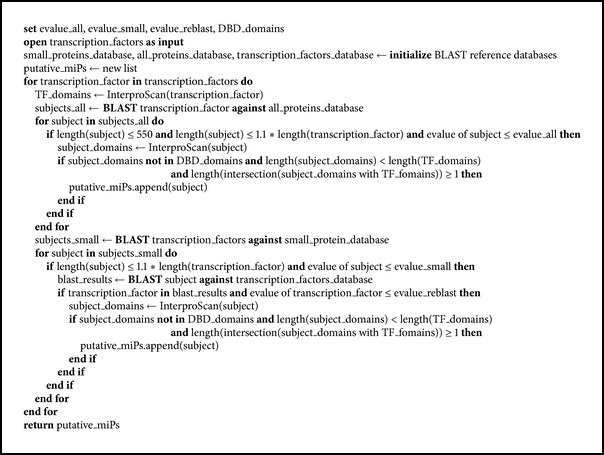
Pseudocode of miP3.
