# Supplementary material for: microProtein Prediction Program (miP3): A Software for Predicting microProteins and Their Target Transcription Factors
Source: Int J Genomics. 2015 Apr 28;2015:734147. doi: 10.1155/2015/734147 (PMC4427850; doi:10.1155/2015/734147)
Supplement: Supplementary file 1 — The Supplementary Material contains two tables. Supplemental Table 1 compares the performance of miP3 with different parameter values. Supplemental Table 2 lists known or putative miPs that were used for performance assessment [file 734147.f1.pdf]

SUPPLEMENTAL DATA

Supplemental table 1. Number of true positives and false negatives based on the 17 characterized miPs (Supplemental table 2) found with different e-value parameters. Highlighted row has the best performance and its parameters were set as default in miP3.

Param 1: e-value all	Param 2: e-value small	Param 3: e-value reblast	true positive	false negative	Recall	Number of predicted miPs
1e-7	0.5	0.1	10	7	0.59	875
1e-6	0.5	0.1	10	7	0.59	891
1e-5	0.5	0.1	10	7	0.59	915
1e-5	0.5	0.1	10	7	0.59	915
1e-6	1	0.1	10	7	0.59	920
1e-5	1	0.1	10	7	0.59	944
1e-7	0.1	0.01	9	8	0.53	770
1e-7	0.5	0.01	9	8	0.53	774
1e-7	1	0.01	9	8	0.53	775
1e-6	0.1	0.01	9	8	0.53	786
1e-6	0.1	0.01	9	8	0.53	786
1e-6	0.5	0.01	9	8	0.53	790
1e-6	1	0.01	9	8	0.53	791
1e-5	0.1	0.01	9	8	0.53	810
1e-5	1	0.01	9	8	0.53	815
1e-6	0.1	0.1	9	8	0.53	815
1e-6	0.1	0.1	9	8	0.53	815
1e-4	0.1	0.01	9	8	0.53	824
1e-4	0.1	0.01	9	8	0.53	824
1e-4	1	0.01	9	8	0.53	829
1e-7	0.5	1e-5	8	9	0.47	718
1e-7	1	1e-5	8	9	0.47	718
1e-7	0.1	1e-5	8	9	0.47	718
1e-7	0.1	1e-5	8	9	0.47	718
1e-7	0.1	1e-4	8	9	0.47	732
1e-7	0.1	1e-4	8	9	0.47	732

1e-7	0.5	1e-4	8	9	0.47	732
1e-6	0.1	1e-5	8	9	0.47	734
1e-6	1	1e-5	8	9	0.47	734
1e-7	0.1	1e-3	8	9	0.47	743
1e-6	0.1	1e-4	8	9	0.47	748
1e-6	0.5	1e-4	8	9	0.47	748
1e-6	0.5	1e-4	8	9	0.47	748
1e-6	1	1e-4	8	9	0.47	748
1e-6	0.1	1e-3	8	9	0.47	759
1e-5	1	1e-5	8	9	0.47	759
1e-5	0.5	1e-5	8	9	0.47	759
1e-5	0.5	1e-5	8	9	0.47	759
1e-6	1	1e-3	8	9	0.47	759
1e-5	0.1	1e-4	8	9	0.47	773
1e-5	0.1	1e-4	8	9	0.47	773
1e-5	1	1e-4	8	9	0.47	773
1e-5	0.5	1e-4	8	9	0.47	773
1e-4	0.5	1e-5	8	9	0.47	774
1e-4	0.1	1e-5	8	9	0.47	774
1e-4	1	1e-5	8	9	0.47	774
1e-4	0.5	1e-5	8	9	0.47	774
1e-4	0.1	1e-5	8	9	0.47	774
1e-4	1	1e-5	8	9	0.47	774
1e-5	0.1	1e-3	8	9	0.47	783
1e-5	1	1e-3	8	9	0.47	783
1e-4	0.1	1e-4	8	9	0.47	787
1e-4	0.5	1e-4	8	9	0.47	787
1e-4	0.5	1e-4	8	9	0.47	787
1e-4	1	1e-4	8	9	0.47	787
1e-4	0.5	1e-3	8	9	0.47	797
1e-4	0.1	1e-3	8	9	0.47	797
1e-4	0.1	1e-3	8	9	0.47	797
1e-4	1	1e-23	6	11	0.35	763
1e-4	1	1e-23	6	11	0.35	763

1e-4	0.1	1e-23	6	11	0.35	763
1e-7	0.5	1e-23	5	12	0.29	691
1e-7	0.5	1e-23	5	12	0.29	691
1e-7	0.1	1e-23	5	12	0.29	691
1e-7	1	1e-23	5	12	0.29	691
1e-6	1	1e-23	5	12	0.29	710
1e-6	1	1e-23	5	12	0.29	710
1e-6	0.1	1e-23	5	12	0.29	710
1e-5	0.5	1e-23	5	12	0.29	738
1e-5	0.5	1e-23	5	12	0.29	738
1e-5	0.1	1e-23	5	12	0.29	738

Suplemental table 2. *A. thaliana* miPs that have been characterized in the literature or that have been tested in yeast two hybrid assays in a previous study [2]. Green: known or putative miPs detected by miP3. Yellow: A known miP not detected by miP3 because the protein sequence is not available in the TAIR database [15]. Red: A known miP not detected by miP3 because it is too divergent in sequence from any TF. Orange: bHLH miP not detected by miP3 because they contain the bHLH domain (IPR011598) but are missing the basic residues upstream of the HLH domain.

Locus name	Previously characterized	Predicted by miP3	Physically interacts	Gene name (Reference)
At1G14760	Yes	Yes		KNATM [6, 7]
AT2G45450	Yes	No		LITTLE ZIPPER 1 [4, 5]
AT3G60890	Yes	Yes		LITTLE ZIPPER 2 [4, 5]
AT3G52770	Yes	Yes		LITTLE ZIPPER 3 [4, 5]
AT2G36307	Yes	No		LITTLE ZIPPER 4 [4, 5]
AT1G74660	Yes	Yes		MINI ZINC FINGER 1 [16]
AT3G28917	Yes	Yes		MINI ZINC FINGER 2 [16]
AT1G18835	Yes	Yes		MINI ZINC FINGER 3 [16]
AT2G42870	Yes	Yes		PHYTOCHROME RAPIDLY REGULATED1 [17, 18]
AT3G58850	Yes	Yes		PHYTOCHROME RAPIDLY REGULATED2 [17, 18]
AT1G02340	Yes	No		LONG HYPOCOTYL IN FR 1 [19]
AT1G74500	Yes	No		ACTIVATION-TAGGED BRI1-SUPPRESSOR 1 [21]
AT3G05800	Yes	No		ATBS1-INTERACTING FACTOR 1 [21]
AT5G39860	Yes	No		PACLOBUTRAZOL RESISTANCE1 [22]
AT1G26945	Yes	No		KIDARI [23]
AT5G07260*	Yes	Yes		LITTLE SIPPER [2]
AT1G09950	Yes	Yes		RESPONSE TO ABA AND SALT 1 [2]
AT1G15215	No	Yes	Yes [2]	Uncharacterized
AT3G19550	No	Yes	Yes [2]	Uncharacterized
AT1G61200	No	Yes	No [2]	Uncharacterized
AT3G19530	No	Yes	No [2]	Uncharacterized
AT1G20280	No	Yes	No [2]	Uncharacterized

*LITTLE SIPPER's locus name was misprinted as AT4G26920 in [2]. AT4G26920 is a paralog of LITTLE SIPPER. Corrections will be published in Plant Physiology.
